# Photon hopping and nanowire based hybrid plasmonic waveguide and ring-resonator

**DOI:** 10.1038/srep09171

**Published:** 2015-03-16

**Authors:** Zhiyuan Gu, Shuai Liu, Shang Sun, Kaiyang Wang, Quan Lyu, Shumin Xiao, Qinghai Song

**Affiliations:** 1 Department of Electrical and Information Engineering, Harbin Institute of Technology, Shenzhen, Guangdong, China. 518055; 2 Department of Materials Science and Engineering, Harbin Institute of Technology, Shenzhen, Guangdong, China. 518055; 3 National Key Laboratory on Tunable Laser Technology, Harbin Institute of Technology, Harbin, China. 158001

## Abstract

Nanowire based hybrid plasmonic structure plays an important role in achieving nanodevices, especially for the wide band-gap materials. However, the conventional schemes of nanowire based devices such as nano-resonators are usually isolated from the integrated nano-network and have extremely low quality (Q) factors. Here we demonstrate the transmission of waves across a gap in hybrid plasmonic waveguide, which is termed as “photon hopping”. Based on the photon hopping, we show that the emissions from nanodevices can be efficiently collected and conducted by additional nanowires. The collection ratio can be higher than 50% for a wide range of separation distance, transverse shift, and tilt. Moreover, we have also explored the possibility of improving performances of individual devices by nano-manipulating the nanowire to a pseudo-ring. Our calculations show that both Q factor and Purcell factor have been increased by more than an order of magnitude. We believe that our researches will be essential to forming nanolasers and the following nano-networks.

Manipulation of light in subwavelength and nanoscale structures is of central importance for the researches on miniaturizations of coherent light sources and highly integrated photonic network^1^,^2^. A number of nanostructures such as nanoparticles^3^ and antennas^4^ have been successfully developed to control the light confinement and emission in subwavelength scale. Nanowires are prominent examples due to their intrinsic advantages, e.g. dislocation free single crystalline, cost-effective synthesis, high index of refraction, and broad range of materials^5^,^6^,^7^,^8^. In past decade, coherent light sources with tiny effective mode areas have been successfully detected from different subwavelength cavities, including ZnS^9^, CdS^10^, GaN^11^, and GaSb^12^ nanowires. These breakthroughs have triggered continuing successes in subwavelength photonic devices with better performances, lower cost, and lower energy consumptions. However, the performances of such devices face severe challenge when their sizes are further reduced to nanoscale. This is caused by two main limitations. The mode volumes of nanowire based devices are usually comparable to their physical dimensions^13^. And the light confinements of nanowires are very poor due to their extremely low end-facet reflections^14^,^15^.

Owing to the better confinement of surface plasmon polaritons (SPP)^16^, the combination of semiconductor nanowire and plasmonic waveguide has been utilized to reduce the effective mode volume (V_eff_)^17^. Soon after the first realization of metal-coated subwavelength laser^18^ and plasmonic bowtie laser^19^, semiconductor-core-metal-shell nanowire^20^ and nanowire-metal hybrid waveguide^21^ have been quickly proposed. Very recently, nanolasers with ultrasmall effective mode volume (or area) have been successfully demonstrated in both CdS^22^ nanowire and GaN^23^ nanowire based hybrid plasmonic waveguides. The successes in ultrasmall mode volumes have boosted a series of potential applications, e.g. ultrasensitive optical sensors and ultrafast directly modulated light sources. However, the obtained Q factors of such structures are usually extremely low due to their small reflectivity at end-facets^22^,^23^. Most importantly, the reported hybrid plasmonic lasers are all insulated devices^22^,^23^ due to the finite lengths of nanowires. To date, the collection or re-collection of output lasers and the interconnection with other nano-devices have not been thoroughly explored. Therefore, developing a simple, robust, and general collecting mechanism has become a key step to develop the nano-network and to improve the performances of individual device.

## Results and Discussion

**Figure 1:**
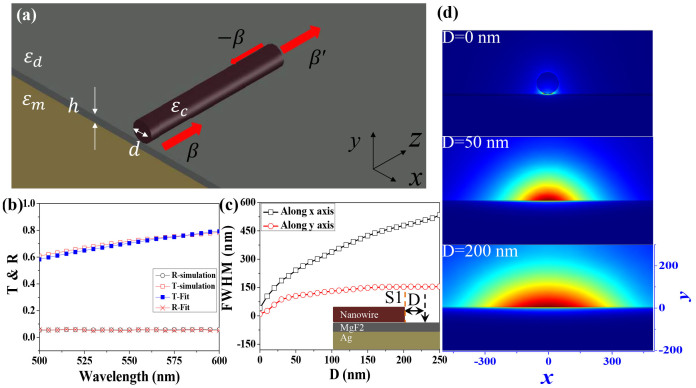
(a) The schematic picture of the hybrid plasmonic waveguide. A semi-infinite long nanowire is placed on a metal substrate insulated by an infinite thin low-index dielectric layer. The end-facet of nanowire is marked as S1. The silver is modeled by Drude model (see supplemental information) and the permittivity of CdS and MgF_2_ are set as *ε_c_* = 5.76 and *ε_d_* = 1.96. (b) The transmittance (*T*, open squares) and reflectance (*R*, open circles) of the waveguide at interface S1. Here *d* = 100 nm and *h* = 5 nm. The theoretical transmittance and reflectance with overlap integral are plotted as solid squares and crosses. (c)The evolutions of FWHM of the output beam in x direction (open squares) and y direction (open circles). (d) The field patterns of propagating mode at 550 nm in the planes with distances *D* = 0 nm, 50 nm, and 200 nm from interface S1.

Prior to pursuing the high collection efficiency and improving the performance, it is worth to understand the main leakages of conventional hybrid plasmonic devices. In a plasmonic waveguide (see Fig. 1(a)), due to the continuity of electric displacement (D, D = εE) along y-direction, the electric field (E) in low-index region is very high and thus most of energy will be stored in the insulating layer, resulting in extremely small effective mode area and long travelling distance^21^,^22^,^23^. Once the propagating waves reach the end-facet of waveguide, the leakages happen. As the effective refractive index of hybrid plasmonic mode is very low (n_eff_ ~ 1.7 for the structure in Fig. 1), the reflectance at end-facet is usually very low and thus gives very small Q factor of conventional Fabry-Perot (F-P) nano-cavity. The interesting phenomenon lies in the transmitted waves. Although the field distributions between hybrid plasmonic mode and conventional plasmonic mode are quite different (see examples in top and bottom panels of Fig. 1(d)), their fundamental light confinements are both dependent on the SPP at the metal-dielectric interface. Consequently, the transmission at end-facet of nanowire or waveguide can be analogue to the transmission between waveguides or fibers with different structural parameters. From this perspective, while scattering light to free space can happen, most of light can be coupled to conventional SPP, which is also well confined at metal-dielectric interface. Thus the outputs from hybrid plasmonic devices can be potentially collected.

To verify above analysis, we then numerically calculated the transmission and reflection of hybrid plasmonic mode at the end-facet. The results are summarized in Fig. 1(b). We can find that the reflectance (open circles) is only ~6% in a wide frequency range, consistent with the small n_eff_. For an F-P cavity with length L = 1 μm, the calculated Q factor is only around 7 (Q = −L*k_a_*/ln|*r*^2^|, where r is the reflection coefficient at end-facet). Consequently, the transmission at the end-facet is very high. The opened squares in Fig. 1(b) show that almost 60%–80% of energy can be detected outside the hybrid plasmonic waveguide.

Besides the high transmission efficiency, the field distributions of output beams have also been studied. As depicted in Fig. 1(c), the full width half maximum (FWHM) of the output beam increases gradually from 40 nm at D = 0 nm to about 530 nm at D = 250 nm along x-axis. The field distribution in the vertical direction is quite different. While the FWHM also increases at the beginning, it becomes saturated at D> 30 nm. From the corresponding field distributions in Fig. 1(d), we thus know that the electromagnetic waves transit from hybrid plasmonic mode to regular SPP along the Silver-MgF_2_ interface, consistent with our above analysis. Then the phenomena in Fig. 1(c) can also be understood. The divergence along x-axis is caused by the disappearance of light confinement in this direction, which induces diffraction as conventional waveguide. The saturation in y-axis is simply formed by the confinement of SPP.

To fully understand the transmission at end-facet, we have also theoretically studied the structure in Fig. 1 by using a mode-matching approach, i.e. overlap integrals^24^. In the theoretical model, the left side and right side of the interface are set as input and output waveguides and the hybrid plasmonic and SPP modes are considered as their fundamental waveguide modes, respectively. By defining the field distributions of input wave, transmitted wave, and reflected waves as 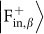, 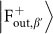, and 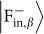 (see the definitions in supplemental information) and considering the continuity of field component along z-axis at the interface, the coupling relation between the hybrid plasmonic mode and SPP mode can be expressed as
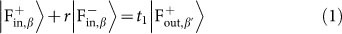
where *t*_1_ and *r* are the transmission and reflection coefficients. To apply orthogonality we multiply equating (1) from the left and from the right by one of the following three operators 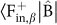, 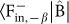, or by 〈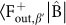 and reduce the results when small reflection is taken into account. Then we can get
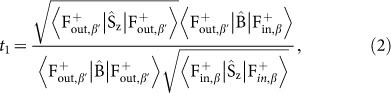

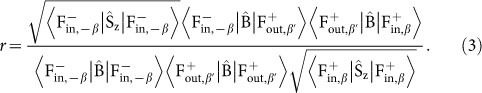
Here we set 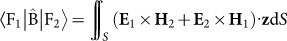 and 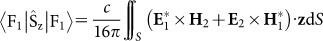 (operators 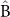 can be found in method). Thus the transmission and reflection coefficients are strongly dependent on the field distribution of the waveguide modes, which can be simply calculated by waveguide theory. By using the mode analysis in finite element method, we have successfully calculated the field distributions of hybrid plasmonic mode and conventional SPP mode and computed the corresponding transmittance and reflectance. The results are also summarized in Fig. 1(b) for a direct comparison. We can see that the theoretical results (solid squares and crosses) and numerical calculation match very well and most of energy transmit to conventional SPP mode.

**Figure 2:**
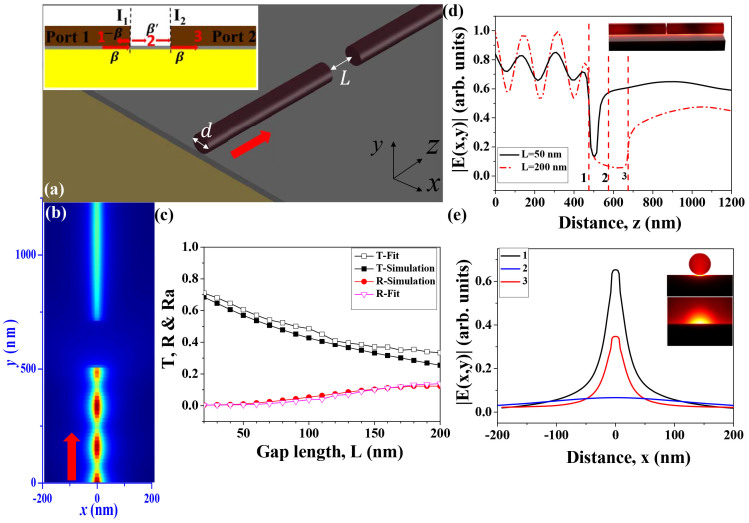
(a) The Schematic picture of two nanowires based hybrid plasmonic waveguide. The separation distance between two nanowires is *L* and the diameters of two nanowires are both d = 100 nm. The other settings are the same as Fig. 1. The inset defines the interfaces and corresponding vectors (β,β', and −β) in different regions. (b) The electric density distribution in MgF_2_ insulating layer. Efficient photon hopping can be directly observed. Here L is 200 nm. (c) The transmittance (*T*, solid squares), reflectance (*R*, red dots) as a function of *L*. The fitted transmittance and reflectance are shown as open squares and open down triangles, respectively. (d) The field distribution (|E_x,y_|) along the line that is 3 nm below the contact line between nanowire and MgF_2_. The gap lengths are L = 50 nm (solid line) and L = 200 nm (Dash dotted line). (e) The field distribution (|E_x,y_|) along x-axis in the insulating layer at position I, II, and III that are marked by vertical dashed lines in (d).The insets in (e) are the corresponding field patterns.

From Eqs. 1–3 we know that the transmission is dependent on the field distributions. Considering the well confinement of SPP mode in y-direction and slow divergence in x-direction (see Fig. 1(c)), the emitted light has the possibility to be collected by placing a receiver nearby the emitting port. For simplicity, we have placed a nanowire with the same size parameters right behind the first one. The structure and the end-facets are schematically shown in Fig. 2(a) and its inset. We have numerically computed the wave propagation inside the three-dimensional structure. Figure 2(b) shows the electric density distribution of hybrid plasmonic mode at λ = 550 nm at the Ag-MgF_2_ interface with gap length L = 50 nm. While a gap appears, we can see that the waves mostly transmit into the second nanowire. Figure 2(c) quantitatively shows the numerical calculated transmission (black solid squares) as a function of gap length. We found that the transmittance maintained at high value even though it gradually decreased with the increase of L. The transmittance to the second nanowire with L = 200 nm is about 25%, indicating high collection ratio can be obtained by such simple design.

The transmittance at L = 50 nm in Fig. 2(c) is around 0.57, which is actually a surprisingly high value. According to the field distributions in Fig. 2(e), the light is well confined as hybrid plasmonic mode in the insulating layer at two nanowire regions. In the gap, its field distribution is close to the conventional SPP in the middle panel of Fig. 1(d). Thus similar to the previous study on the propagation of SPP across narrow grooves in silver film^25^,^26^,^27^,^28^, the transmitted waves experience a transition from hybrid plasmonic to regular SPP and a transition back to hybrid plasmonic. And the total transmittance can also be computed with overlap integral. Without considering the interference, the total transmittance through two interfaces should be the product of the transmittances at two interfaces. Similar to Eq. (1), the coupling from SPP to hybrid plasmonic mode at end-facet I_2_ can be described as
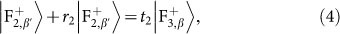
where 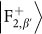 and 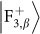 represent the field distributions of propagating waves in regions 2 and 3. Combined with Eqs. 1–3, the calculated total transmittance at λ = 550 nm is T = |t_1_^2^|*| t_2_^2^| ~ 0.495, which is far below the numerically computed value. Therefore, a new mechanism must be considered besides the simple collection.

It is quite intuitive to consider the interference effect between two parallel interfaces I_1_ and I_2_. As we know, once the phases between reflected waves from two interfaces have π shift, the reflectance will be decreased and the transmittance is increased. To verify such hypothesis, we have studied the value of |E_x,y_| along the line that is 3 nm below the contact line between nanowire and MgF_2_. The results are shown in Fig. 2(d). While obvious interference can be observed within Z = 0–400 nm, the peak positions at L = 50 nm and L = 200 nm are almost the same, indicating the main reflection is caused by the interface I_1_ and the interference effect can be excluded here. To further support this conclusion, we have also studied the influences of the end-facets' shape. Our calculation shows that convex end-facet won't affect the total transmission significantly (see supplemental information).

As indicated by Eqs. 1–4, the field distributions can be another issue to affect the transmission and reflection. If the field distribution is localized more close to the hybrid plasmonic mode, the total transmission should be increased. While the field distribution inside the gap is close to conventional SPP, the details are quite different. According to Fig. 1(c), the transmitted waves diffract in both x and y directions. Then the component E_z_ appears. Along the z axis, the electric displacement D_z_ should be continuous. Similar to the light confinement in hybrid plasmonic mode, the electric field (|E_z_|) in the air gap should also be significantly increased due to capacitive energy storage at CdS-air-CdS structure. This has also been verified by calculating the distribution of both |E| and |E_z_| along the axle center of nanowire (see supplemental information). Therefore, the total field distribution of SPP mode inside the air gap is modified and is more close to the initial distribution, giving an improved transmittance (or collection ratio at the second nanowire).

This hypothesis has also been further proved by the theoretical calculation following Eqs. 1–4. By employing the calculated field distributions at the cross-sections close to two interfaces instead of typical SPP modes from mode analysis, the total transmittance have also been computed. The transmittance and reflectance are plotted as open squares and opened down triangles in Fig. 2(c) too. We can find that the transmission also decreases with the increase of gap length and the calculated transmission and refection efficiencies match the full-wave numerical simulation well. Such a big difference between the computed transmissions by overlap integral has further proved the importance of field distribution for efficient collection. With the increase of gap length, the capacitive energy storage decreases the divergence of SPP mode increase. Thus the total transmittance also reduces.

**Figure 3:**
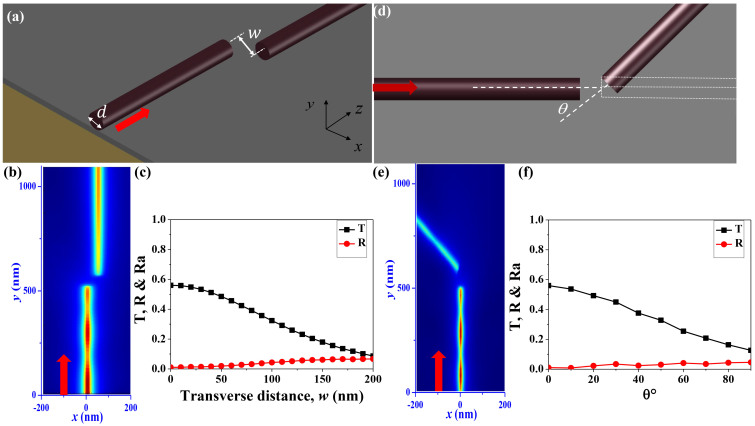
The robustness of the light collection. (a) and (d) depict the transverse shift (w) and tilt angle (θ) of the second nanowire. (c) and (f) are the dependences of *T*, and *R* on w and *θ*, respectively. (b) and (e) show the field (|E_x,y_|) distributions with *w* = 50 nm and *θ* = 50 degree.

Placing two nanowires end by end is of course possible in nano-manipulation^13^. However, it is extremely difficult to arrange their axles in a single line as Fig. 2. To explore the light collection for real applications, we have numerically studied the dependences of transmittance on the transverse shift along x-axis (see Fig. 3(a)). The results are summarized in Fig. 3(c). With the increasing of *w*, the transmittance decreases slowly. When *w* = 100 nm and gap length *L* = 50 nm, the transmittance is still around 32%. This means that the collection efficiency can be very high by placing two nanowires end by end with slight overlap. Similarly, the transmittance *T* is also quite robust to the tilt of the second wire, which is also possible in nano-manipulation. When the tilt angle *θ* ≤ 40 degree (initial gap length L is also 50 nm), the transmittance is higher than 39.5% in Fig. 3(f)). The examples of field patterns in MgF_2_ layer are plotted in Figs. 3(b) and 3(e). Similar to Fig. 2, the transmitted light follows the same hybrid plasmonic mode as the incident one. Thus the transmission follows Eqs. 1–4 well. With the increase of transverse shift and tilt, the capacitive energy storage gradually decreases. Consequently, the total transmission (or collection ratio) degrades. Moreover, we have also tested the influence of material dispersion and anisotropy of nanowires. Our results show that the dispersion and anisotropy do not affect the total transmittance obviously (see supplemental information). And the cross-sectional geometries of the waveguide also have been investigated (see supplemental information). Both triangle and hexagonal cross-sections show similar transmittance as the results of circular cross-section in Fig. 2. Therefore, we know that the high collection ratio can be obtained in large ranges of gap length, transverse shift, tilt, material dispersion, shapes of end-facet, and geometries of cross-section, making it to be quite robust in practical applications. Then it is possible to form a hybrid plasmonic nano-network.

### Hybrid plasmonic ring-resonators

**Figure 4:**
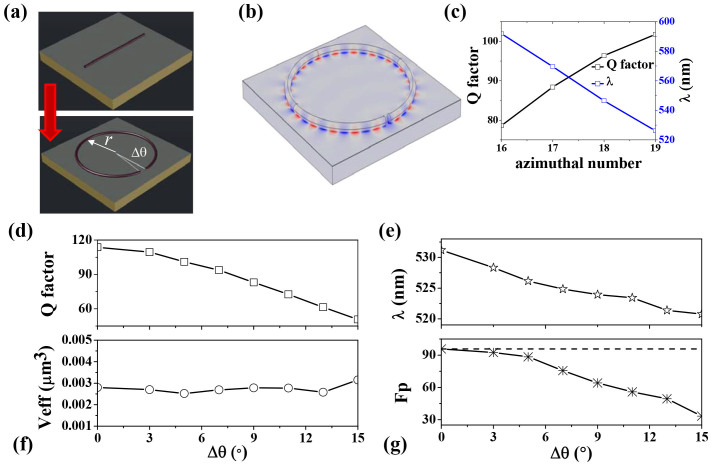
(a) Schematic picture of hybrid plasmonic ring-resonator. The size parameters of ring resonator and nanowire are *r* = 1000 nm and *d* = 100 nm, respectively. And the separation between two end-facets is determined by *Δθ*. (b) The field pattern of resonance at λ = 527 nm. Here *Δθ* = 5° and the Azimuthal number m is 19. (c) The Q factors and resonant wavelengths of different resonances. (d)–(g) The Q factor, resonant wavelength, effective mode volume, and Purcell effect of the mode with m = 19 as a function of Δθ.

In additional to form a potential anno-network, we can also utilize the high collection ratio to improve the performance of individual device. Different from the bulk materials, nanowires usually have quite different physical properties. One of them is the possibility in tailoring the shapes of nanowires. In general, nanowires are cut with micropositioning system and placed on silver substrate with a 5 nm magnesium fluoride (MgF_2_) layer^22^,^29^. They can be pushed by tungsten needle on piezoelectric stage to face to face or side by side and form a ring-like structure^13^,^29^. And the corresponding resonant properties will be quite different. One example is shown in Fig. 4(a), where the shape of nanowire follows a perfect ring and the separation distance between two end-facets is defined as a *Δθ*. Then the possibility of forming long-lived resonance turns to be very clear. While the waves have high transmittance at one end-facet, the transmitted light can be efficiently collected by the same nanowire at the other end-facet. Then both the transmitted waves and reflected waves are still well confined within the cavity and only a small portion of light is scattered into free space. Consequently, the light confinement in pseudo-ring should be very close to that in perfect ring resonator. Figure 4(b) shows an example of the resonance in the nanowire-based ring-resonator. From the field distribution within the MgF_2_ insulating layer, we can see that a whispering gallery (WG) like mode with Azimuthal number m = 19 has been formed. Similar to our expectation, the calculated Q factor is still about 100, which is more than an order of magnitude higher than the Fabry-Perot cavity^22^. One may argue that the Q factor of this ring resonator is not as good as regular ring resonator with similar size^29^. We note it has significant advantages in both effective mode volume (V_eff_) and l Purcell factor (F_p_). For the mode in Fig. 4(b), the calculated V_eff_ is about 3 × 10^−3^ μm^3^. Then the corresponding Purcell factor (Fp = 3Q(λ/n)^3^/(4π^2^V_eff_)) can be as high as 90. Both V_eff_ and F_p_ are orders of magnitude higher than regular dielectric cavity^30^,^31^ and conventional F-P hybrid plasmonic cavity, indicating the potential applications in nanosensors and nano-cavity quantum electrodynamics.

The resonance in Fig. 4(b) is not a special mode that generate high Q factor like the modes around avoided resonance crossing^32^,^33^. This calculated WG-like modes with different Azimuthal number in Fig. 4(c) shows that the high Q resonance is quite general in the nanowire based ring-resonator. Similar to Fig. 2, the high Q factor is also robust to the changes on separation gap, making the new design to be easily realized. Figures 4(d)–(g) show the dependences of Q factor, resonant wavelength, effective mode volume, and Purcell factor on the size of separation distance *Δθ*. With the increase of *Δθ* (size of air gap), the resonant wavelength shifts to blue side in Fig. 4(e). According to Figs. 2 and 3, the collection ratio of the nanowire also decreases, generating a reduction in Q factor in Fig. 4(d). When *Δθ* is smaller than 5 degree, which corresponds to a gap size *Δθ* × *R* about 87 nm, the reduction is very slow and the Q factor is very close to that of a perfect ring resonator with *Δθ* = 0. The reduction of Q factor increases at larger *Δθ*. But the Q factor at *Δθ* = 13 degree (gap size ~ 226 nm) is still around half of the value of perfect ring. Meanwhile, as the main and maxima fields are confined within the hybrid plasmonic region, the effective mode volume (V_eff_) is almost a constant around 0.003 in Fig. 4(f). Then the behavior of Purcell factor in Fig. 4(d) follows the change of Q factor. The value of F_p_ is still as high as 50 even at *Δθ* = 13 degree (gap size ~ 226 nm). Therefore, simply tailoring the nanowire to a ring-like hybrid plasmonic resonator can be an effective way to achieve relative high Q, ultrasmall mode volume, and larger Purcell factor simultaneously in nanoscale.

It is important to note that perfect hybrid plasmonic ring-resonator without air gap has been thoroughly studied before. The transverse cross-sections in some studies are the rectangles^34^,^35^, which is supposed to be fabricated with top-down etching. However, the nanoscale circular devices are extremely difficult to be fabricated even with E-beam lithography. Moreover, the etching faces sever challenge in wide bandgap materials such as GaN, where the surface roughness is too large to keep the light confinement^36^. Perfect ring-resonator with circular cross-section has also been proposed very recently^37^. But the nanowire ring without air gap is almost impossible to be synthesized as conventional nanowire. In our design, the ring resonator is formed by manipulating the shape of nanowire^13^,^29^ and thus is much easier to be realized.

**Figure 5:**
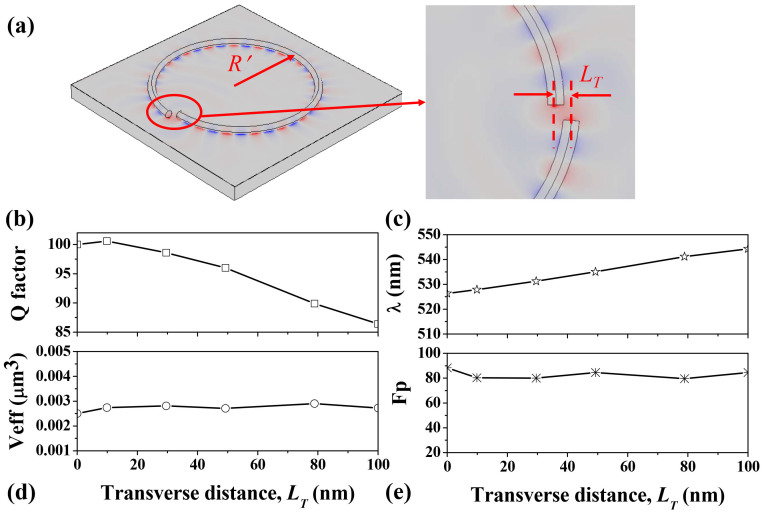
(a) Schematic of hybrid plasmonic spiral shaped ring-resonator with transverse separation *L_T_* and fixed separation angle *Δθ′* = 5°. The field pattern is the calculated WG-like mode with *L_T_* = 100 nm. The Azimuthal number is m = 19. (b)–(e) The Q factor, resonant wavelength, effective mode volume, and Purcell effect of the mode with m = 19 as a function of transverse separation *L_T_*. Here the separation angle *Δθ′* = 5°.

We note that that the key point for the high Q resonance is the photon hopping across the air gap instead of the cavity shape. This is extremely important since the perfect ring-like shapes are impossible to be obtained experimentally. One example is the spiral shaped cavity as shown in Fig. 5(a), whose radius (*R′*) is described as *R′ = R(1 + εθ/2π)*. Here we set *R* = 1 μm and *ε* is the shape deformation. The transverse distance between two end-facets is defined as *L_T_* = *R × ε* and the fixed separation angle is *Δθ′* = 5 degree. Compared with the structure in Fig. 4, the spiral shape introduces an additional lateral shift, which usually exists in real experiment. In Figs 2 and 3, we have shown that the photon hopping is quite robust to the lateral shift. Thus we can expect that the Q factor might also be maintained in the spiral shaped ring resonator. As shown in Fig. 5(a), WG like mode with Azimuthal number m * = * 19 is formed when the lateral shift is *L_T_* = 100 nm. Our calculations show that the V_eff_ and Q factor are still around 3 × 10^−3^ μm^3^ and 86, respectively. And the corresponding Purcell factor is around 80. Figures 5(b)–5(e) show the Q factor, resonance wavelength, effective mode volume and Purcell factor as a function of transverse distance *L_T_*. Longer transverse distance means larger loss which results in monotonically decreasing of Q factor with the increase of *L_T_*. The effective mode volume doesn't show obvious changes. Meanwhile, as the resonant wavelength increases, Purcell factor also fluctuates slightly around 80. Thus we know that the formation of long-lived resonance by tailoring nanowires is quite robust in real experiments, consistent with the robustness of the photon hopping in Fig. 3. Besides the spiral shaped cavity, we have also obtained similar high Q resonances in other types of deformed ring resonators [see Supplementary Information]. Moreover, the possibility of forming a pseudo-ring by placing the nanowire side by side has also been studied. We found that the WG mode with m = 19 could also have comparable Q factor and Purcell factor as Fig. 4 [see supplemental information]. This structure and the design in Fig. 5 also give the possibility to collect the emission. Similar to the studies in Figs. 2 and 3, the emitted light can be efficiently coupled to other nano-devices by placing a nanowire or a waveguide nearby the outer end-facts.

## Conclusion

In Summary, we have studied the transmission of hybrid plasmonic modes across a small gap. Our numerical results and the theoretical calculation based on overlap integral have demonstrated that high collection ratio can be achieved by placing a second nanowire nearby the emitting end-facet. The collection efficiency is found to be robust to gap length, transverse shift, tilt, material dispersion, and the shapes of end-facets. This finding makes the interaction between isolated nano-devices to be possible and will advance the progress of nano-network. Moreover, since only small portion of light is scattered to free space, we have also proposed a pseudo-ring cavity. Compared with the conventional F-P like hybrid plasmonic cavity, the Q factor and Purcell factor have been dramatically improved by more than an order of magnitude. As both the synthesis of nanowire and the nanoscale manipulation have been widely explored, our findings will significantly expand the applications of nanowires in active devices such as nanolasers and will also boost the researches of nano-network.

## Methods

The mode properties of hybrid plasmonic waveguide and ring-resonator are numerically investigated by the finite element method (COMSOL MULTIPHYSICS 4.4).

### Hybrid plasmonic waveguide

The eigenvalue solver of COMSOL is used to find modes of the hybrid plasmonic waveguide. The effective index n_eff_ is determined from real part of the eigenvalue. Numerical ports are employed to evaluate the transmission and reflection of the waveguide with all simulation domain surrounded by scattering boundary conditions (SBCs).

### Overlap integrals

The definition |F*_β_*〉 should be understood in a sense of 4-component vector of the transverse electromagnetic field:
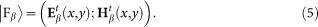


In the case of a waveguide with absorbing dielectrics, the products E* × **H** in orthogonality relation between modes should be modified as **E** × **H**. The normalization operator 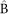 is defined as 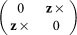.

### Ring-resonator

The whispering gallery like mode with azimuthal number *m* in cylindrical coordinates can be described as
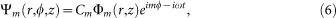
where *ω* is angular frequency, *C_m_* is the coefficient and Φ*_m_*(*r*, *z*) denotes the field profile at the cross section. The expression Φ*_m_*(*r*, *z*) should satisfy the character equation for a given *m*
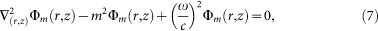
In this equation, *ε* is the relative permittivity and *c* is the light velocity in vacuum. The eigenfrequency of the ring resonator is also calculated by eigenvalue solver.

The Q factor is defined as *Q* = *f_Re_*/2*f_Im_*, where *f* = *f_Re_* + *i* * *f_Im_* is the complex eigenfrequency of the mode. And the mode volume is defined as the ratio of the total mode energy and the peak energy density, which is determined by
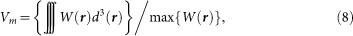
where *W*(***r***) is energy density and takes the form
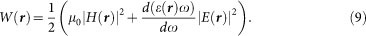


## Supplementary Material

Supplementary Information Supplemental information
