# Supplementary material for: Photon hopping and nanowire based hybrid plasmonic waveguide and ring-resonator
Source: Sci Rep. 2015 Mar 16;5:9171. doi: 10.1038/srep09171 (PMC5378941; doi:10.1038/srep09171)
Supplement: Supplementary Information — Supplemental information [file srep09171-s1.pdf]

Supplementary information

Photon hopping and nanowire based hybrid plasmonic waveguide and ring-resonator

Zhiyuan Gu[1], Shuai Liu[1], Shang Sun[2], Kaiyang Wang[1], Quan Lv[1], Shumin Xiao[2, †], Qinghai Song[1, 3,*]

[1]Department of Electrical and Information Engineering, Harbin Institute of Technology, Shenzhen, Guangdong, China, 518055, [2]Department of Materials Science and Engineering, Harbin Institute of Technology, Shenzhen, Guangdong, China, 518055, [3]National Key Laboratory on Tunable Laser Technology, Harbin Institute of Technology, Harbin, China, 158001

E-mail: *qinghai.song@hitsz.edu.cn

†shuminxiao@gmail.com

Drude model

The dielectric function of silver is described by Drude model:

$$\varepsilon_\omega = \varepsilon_\infty - \omega_p^2/\omega(\omega + i\gamma).$$

Here $\varepsilon_\infty = 3.5$, the plasma frequency $\omega_p = 1.33 \times 10^{16}$Hz, and collision frequency $\gamma = 2.75 \times 10^{13}$Hz.

Crystal anisotropy of CdS nanowire:

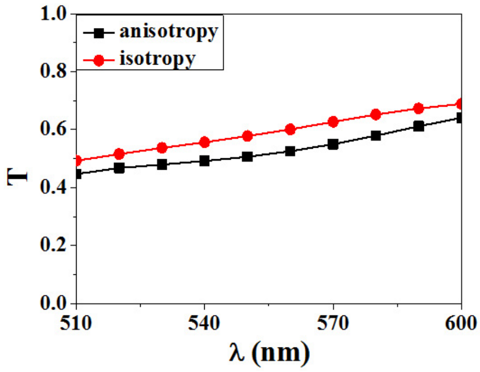

Figure S1. Transmittance of hybrid plasmonic waveguide as a function of wavelength. Red dots represent the transmittance of waveguide without crystal anisotropy. And black squares represent transmittance of waveguide with isotropy. Here we fix the separation between two nanowires as 50 nm.

The dielectric constant of CdS nanowire in the main text is ε = 5.76, which is considered to be isotropic as the definition in Ref-22 (Oulton R. F. et al, *Nature* **2009**, 461, 629-632). However, the real CdS nanowire is usually anisotropic and dispersive. Here we show that the anisotropy and dispersion of CdS won't change the results significantly. The dielectric constant tensor of anisotropic material is described as $\vec{\varepsilon} = \begin{bmatrix} \varepsilon_1 & 0 & 0 \\ 0 & \varepsilon_2 & 0 \\ 0 & 0 & \varepsilon_3 \end{bmatrix}$. For dielectric materials,$n_a = \sqrt{\varepsilon_1}$, $n_b = \sqrt{\varepsilon_2}$ and $n_c = \sqrt{\varepsilon_3}$. For single crystal, we set $\varepsilon_1 = \varepsilon_2 \neq \varepsilon_3$ and $n_a = n_b = n_o$, and $n_c = n_e$. Here n_o and n_e are refractive indexes of the ordinary light and extraordinary light. Besides the crystal anisotropy, the material dispersions have also been considered. The dispersive formulas of the ordinary light and extraordinary light are $n_o^2 = 5.1792 + \frac{0.23504}{\lambda^2 - 0.083591} + \frac{0.036927}{\lambda^2 - 0.23504}$ and$n_e^2 = 5.2599 + \frac{0.20865}{\lambda^2 - 0.0.10799} + \frac{0.027527}{\lambda^2 - 0.23305}$, respectively. The simulated results (black squares) are shown in

Fig. S1, where the transmittance without anisotropy and dispersion are also plotted (red dots). We can see that the transmittance only decreases by 5% when both crystal anisotropy and material dispersion are considered. Our detail analysis shows that the main contribution comes from the material dispersion. Thus we know that the crystal anisotropy and material dispersion only slightly affect the photon hopping effect.

Impacts of end-facet and cross-section of nanowire

Considering the actual situations in real experiments, some nanowires may not have flat end-facets as Figs. 2 and 3. Thus it is also interesting to study the possible influences of the shapes of end-facets. Here we add a convex section with controllable thickness *t* on the end-facets to study the corresponding photon hopping [see Fig. S2(a2)]. The results are plotted in Fig. S2(b). We can see that the transmittance T_S gradually decreases from ~75% to 35% when *S* increases from 40 nm to 160 nm. All these changes are very similar to the results with clean end-facets such as Fig. 2. For a direct comparison, the transmittances of structures in Figs. S2(a) and (c) have also been plotted in Fig. S2(b). While three lines are very close, we can still see that T_S is higher than T_L but is lower than $T_{L'}$. By fitting the separation distance at S = 150 nm, we have also changed the convex thickness t and studied the detail impacts of the convex shapes. As shown in Fig. S2(c), the transmittance goes up slowly with the increase of *t*. It increases almost by 12% when *t* changes from 5 nm to 50 nm. This is also similar to the difference between T_S and $T_{L'}$ in Fig. S2(b). The convex shape of end-facet is more close to slight decrease of the separation distance. Thus we know that the convex shapes of end-facets won't significantly affect the photon hopping efficiency. Moreover, the convex end-facets cannot form the F-P like interference as flat interfaces. Here the high transmittance can also exclude the influence of interference on the total transmittance in Fig .2(c) of the main text.

In additional to the shapes of end-facet, it is also interesting to study the influence of the cross-section. Due to the crystalline lattice, some nanowires have hexagonal and triangular cross-sections. Here will show that the photon hopping holds true in these nanowire too. As shown in Fig. S2(d), the transmission lines of

hexagonal and triangle nanowires are almost the same that of the circular one. Thus we know that the impact of cross-sectional geometries of nanowires on the photon hopping can be negligible.

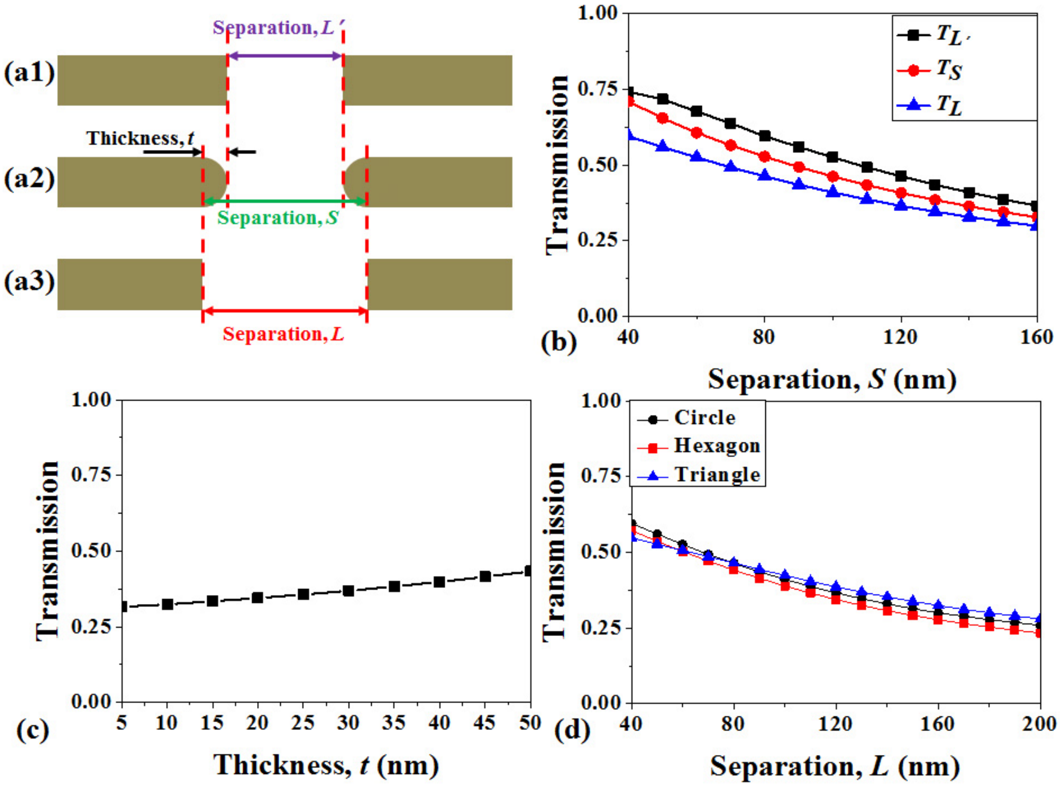

Figure S2. (a1)-(a3) The Schematic pictures of nanowire arrangements with separation distance L', S, L, respectively. (b) $T_{L'}$ (black squares), T_S (red dots) and T_L (blue triangles) represent the transmission of three hybrid plasmonic waveguides (a1-a3) with separation distance L', S, L as a function of S, where separation distances satisfy $L' = S - 2t$ and $L = S$. Here the convex thickness t = 20 nm. (c) The transmission of hybrid plasmonic waveguide with convex nanowires facets (a2) as a function of convex thickness t. Here the separation distance S = 150 nm. (d) The transmission of hybrid plasmonic waveguides (a3) with circular (black dots), hexagonal (red squares) and triangular (blue triangles) cross-section geometries as a function of separation L, respectively.

Field distribution in nanowire

In the main text, we have claimed that the field distributions within the gap area have been redistributed by the capacitive energy storage. To support this information, here we studied the field distribution (|E| and $|E_z|$)

in the gap area. The capacitive energy storage of E_z can be understood by the continuity of D_z of divergent waves inside the gap area. As the $D_{CdS} = D_{air}$ along z-direction, E_{air} is much larger than E_{CdS}. Thus the field within the gap area will be increased and the total transmittance is also affected. Figure S3 shows the $|E_z|$ and $|E|$ along the axes of nanowire in z-direction. We can see that both $|E|$ and $|E_z|$ show similar enhancement within the gap.

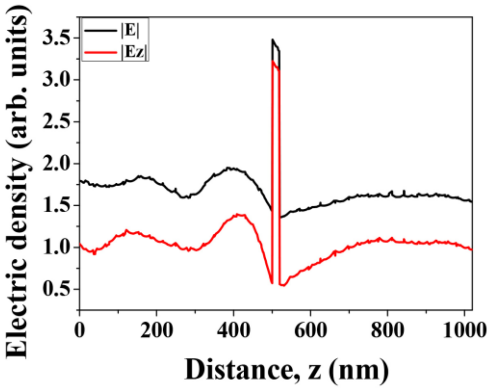

Figure S3. Distribution of |E| (black line) and |Ez| (red line) along the axle center of nanowire. Here the gap length is 20 nm.

Deformed ring resonator:

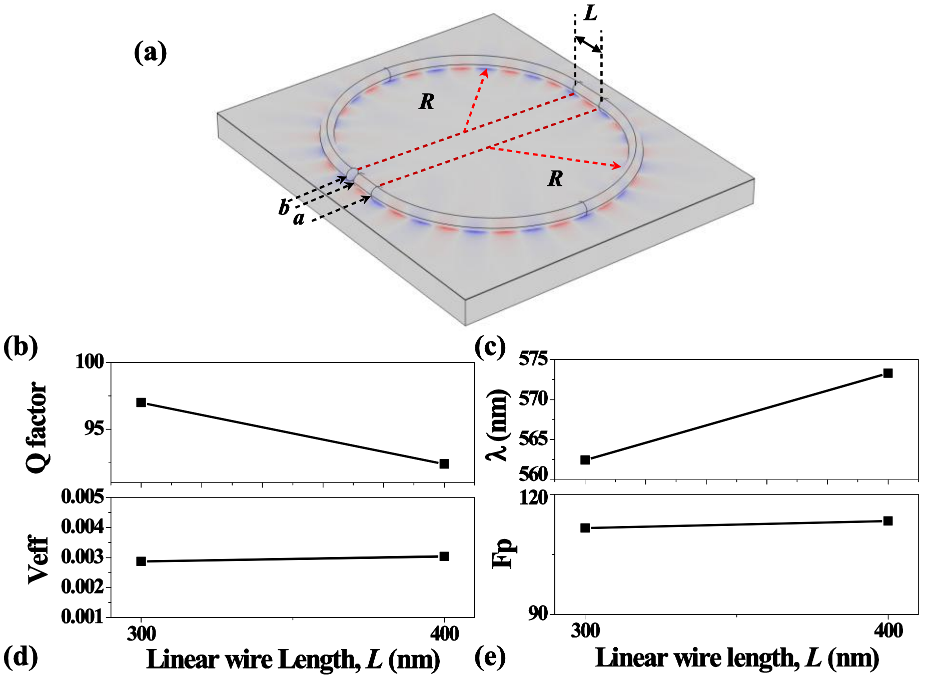

Figure S4. (a) The field pattern of resonance at λ =562 nm of hybrid plasmonic stadium-resonator. Here the size

parameters of resonator and nanowire are L = 300 nm, a = 200 nm, R = 1000 nm and d = 100 nm, respectively. The separation between two end-facets is b = 87.2 nm ($\Delta\theta \approx 5^o$), which is invariant. And the Azimuthal number m is 19. (b) – (e) are the Q factor, resonant wavelength, effective mode volume, and Purcell effect of the mode with m = 19 as a function of L.

As we mentioned in the main text, our new mechanism is not sensitive to the cavity boundary. For example, we have deformed the cavity to a stadium, which is formed by two semicircles and linear nanowires with length L (see the schematic picture in Figure S4(a)). Here we fix the separation between two end-facets as b = 87.2 nm *(*$\Delta\theta \approx 5^o$*)* and a=L-b. Figure S4(a) shows the field pattern of resonant mode. Similar to the results in circular cavity, here the main field is also confined by the hybrid plasmonic mode. Figures S4(b)-(e) illustrate the dependences of Q factor, resonant wavelength, effective mode volume, and Purcell factor on the wire length *L*. In Figure S4(d), the effective mode volume is around 0.00252 μm^3. This value is almost the same as the pseudo-ring resonator in Figure 4(f), showing the independence of the resonant properties on cavity shape. Similar phenomenon also holds true for the Purcell factor (see Figure S4(e) and Figure 4(g)). The Q factors of stadium cavity are also similar to the ring resonator. Slight differences are caused by the increase of the cavity length (intrinsic loss).

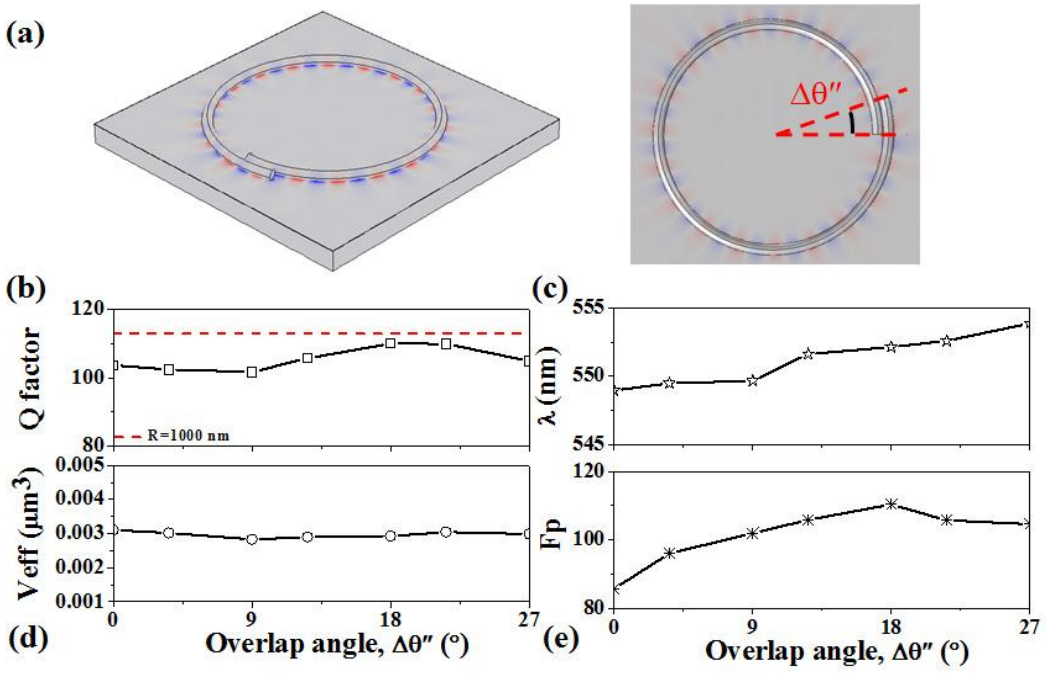

Figure S5 (a) Schematic of hybrid plasmonic spiral ring resonator with overlap angle Δθ″. The radius of the resonator is depend on $R'=R(1+\varepsilon\theta/2\pi)$. Here R = 1 μm and ε = 0.1. Field distribution of WG like mode is an example of resonance with $\Delta\theta''$ = 18 ° and Azimuthal number m = 19. Red dashed line represents the Q factor of perfect ring resonator with R = 1000 nm. (b)-(e) show the dependences of Q factor, resonance wavelength, V_{eff} and Fp on the size of overlap angle $\Delta\theta''$.

In additional to push a nanowire and form a ring resonator with an air gap, it is also possible to place two ends side by side under micromanipulation. Here we would also like to discuss the possibility of generating relative large Q factor and Purcell factor in such a cavity. The schematic picture is shown in Fig. S5, where the radius (R') of resonator is still described as $R'=R(1+\varepsilon\theta/2\pi)$ and the overlapped part is defined as an angle $\Delta\theta''$. For simplicity, we fixed R = 1 μm and ε = 0.1 here. The field pattern in Fig. S5(a) shows that WG-like resonance can still be formed. The corresponding Q factor is around 113, which is even close to the perfect ring without air gap and later shift (see the dashed in Fig. S5(b)). Figures (b)-(e) show the dependences of Q factor, resonant wavelength, effective mode volume, and the Purcell factor on the overlapping between two ends. We can see that simply pushing two ends side by side can also form very nice WG-like resonances. The

formation of high Q and larger Purcell factor in Fig. S5 is also not surprising. Different from the photon hopping across the air gap, here the photon hopping happens between two hybrid waveguides. This kind of energy dissemination is also known as mode coupling in the researches on optical waveguide. Such coupling is dependent on the overlapping distance. This can be seen from the fluctuation of Q factor in Fig. S5(b). Similar to pushing two ends of nanowires face by face, tailoring their positions to side by side can also be an effective way to conduct the emission to other integrated system or to improve the performance of single device.
